# Editorial: Current treatment strategies and integrative medicine for management of pain in sickle cell disease

**DOI:** 10.3389/fpain.2025.1621147

**Published:** 2025-06-10

**Authors:** Kimberlei A. Richardson, Gabrielle Lynn McLemore, Keesha Powell-Roach, Kalpna Gupta

**Affiliations:** ^1^ Department of Pharmacology, Howard University College of Medicine, Washington, DC, United States; ^2^ Department of Biology, School of Computer, Mathematics and Natural Sciences, Morgan State University, Baltimore, MD, United States; ^3^ Division of Nursing Science, School of Nursing, Rutgers University, New Brunswick, NJ, United States; ^4^ Division of Hematology/Oncology, Department of Medicine, School of Medicine, University of California-Irvine, Irvine, CA, United States

**Keywords:** chronic pain, sickle cell disease, integrative medicine, alternative and complementary therapy, treatment strategies, pharmacogenomics, patient-centered care

**Editorial on the Research Topic**
Current treatment strategies and integrative medicine for management of pain in sickle cell disease

This editorial presents a series of articles from a special issue of the Frontiers in Pain Research Journal titled “Current Treatment Strategies and Integrative Medicine for Management of Pain in Sickle Cell Disease.” Sickle cell disease (SCD) is a globally prevalent, inherited condition characterized by rigid, abnormally shaped red blood cells. This disease can lead to vaso-occlusive crises, hemolysis, pain, and other complications. The acute and chronic pain experienced by individuals with SCD is often reported as a debilitating symptom that profoundly impacts their quality of life. Despite numerous efforts to develop more effective treatments, managing pain in this population remains difficult. This collection of five articles underscores the potential of integrative and precision medicine approaches to improve pain management, while revealing the biological, clinical, and systemic components that shape SCD pain experiences and treatment outcomes. In this editorial, the major findings of the various authors involved have been summarized and placed within a broader context while also highlighting the challenges that still lie ahead for the management and treatment of chronic pain in SCD.

Conventional pain management for SCD has depended on opioids, despite their high potential for addiction. Non-opioid, integrative medicine strategies (IMS) show promise as potentially safe and efficacious alternatives for managing SCD pain, but the lack of evidence-based and guideline-recommended strategies for implementing these therapies hinders their widespread use. A critical review of these strategies by Smith et al. highlighted that the full implementation of pharmacological/biobehavioral pain strategies that target mechanistic pain pathways is challenging due to limited knowledge and inadequate resources, including financial support and personnel. The authors recommended personalized medicine, pharmacogenomics, and IMS as potential strategies for managing SCD pain. They updated Melzack's Neuromatrix pain model, and presented a framework for classifying SCD pain subphenotypes and mechanisms, selecting personalized multimodal treatment strategies, and identifying research gaps for potential exploration. The authors contended that precision medicine and integrative health offer a cogent research approach for multimodal, individualized strategies to diagnose and treat acute/chronic SCD pain. Building upon established integrative medicine knowledge, further advancements in metabolic interventions have demonstrated the potential to improve patient outcomes.

SCD disrupts oxygen transport and is characterized by oxidative stress and endothelial dysfunction, which are often exacerbated by hemolysis-induced L-arginine depletion. L-arginine is essential for nitric oxide synthesis, which in turn regulates vascular health and oxidative stress. Recent findings from Christian et al. (2024) in Kinshasa, Democratic Republic of Congo, demonstrated the impact of arginine supplementation on the levels of lactate dehydrogenase, a key marker of hemolysis, in a retrospective cohort of 31 patients with SCD across three different treatment phases. These results highlighted the potential of L-arginine supplementation to mitigate hemolysis-related complications in SCD, as significant reductions in hemolysis-related complications were observed when arginine was combined with hydroxyurea therapy. While arginine supplementation targets physiological mechanisms, other approaches, such as Traditional Chinese Medicine (TCM), take a more holistic approach to address the physical and systemic aspects of SCD care.

Wang et al. sought to integrate TCM principles to guide the use of acupuncture and other alternative strategies, in treating comorbidities related to SCD care. The use of TCM diagnoses introduces a culturally resonant and holistic dimension to categorizing SCD pathologies as syndromes such as Qi and blood deficiency, stagnation, or mixed presentations. Based on these pathologies, patients with SCD were sorted into three different TCM Syndrome groups: Deficiency, Stagnation, and Equal. These groups did not differ in quantitative sensory testing results, but differences were observed in the frequency of vaso-occlusive crises and opioid consumption, and patients' pain profiles. Patients in the Stagnation group experienced heightened pain interference and nociplastic pain, while those in the Deficiency group faced profound chronic pain and comorbidities. These findings highlight the clinical relevance of TCM diagnoses and highlight the potential of TCM-based diagnostics to inform personalized acupuncture and integrative therapies, not only for SCD but also for other clinical populations with complex pathologies. A focus on holistic approaches is critical to long-term patient outcomes and extends to identifying and mitigating skeletal/bone complications associated with SCD.

Bone pain is one of the most pervasive and debilitating complications of SCD, significantly affecting quality of life. Gollamudi et al. provided a comprehensive overview of the mechanisms driving bone complications in SCD, drawing parallels with findings from non-SCD models to elucidate common pathways. By exploring the dynamic interactions between the skeletal and peripheral nervous systems, their review underscored the importance of understanding the nuances of these processes to develop effective, targeted therapies. Furthermore, it highlighted the need for rigorous research to address gaps in our knowledge about the roles of stress erythropoiesis, avascular necrosis, and inflammation in SCD-related bone complications. As we move toward innovative approaches, this work aims to inform future basic and translational research that can ultimately reduce the burden of bone pain and improve outcomes for individuals living with SCD. As bone-related complications highlight the interplay of systemic and cellular mechanisms, genetic research provides deeper insights into individual variability in SCD pain experiences.

Genetic variation has long been hypothesized to influence individual pain perception and severity. Despite this, the relationship between single-nucleotide polymorphisms (SNPs) and SCD-related pain remains underexplored, leaving significant gaps in our understanding and limiting the potential of precision medicine approaches. A systematic review by Gehling et al. synthesized two decades of scientific literature examining the association between SNPs and SCD-related pain outcomes. Significant gaps remain in our understanding of chronic pain phenotypes and their genetic basis. The evidence reviewed in this study points to the potential of genetic variation as a contributor to both acute and chronic pain phenotypes in SCD. However, the field is still in its infancy, requiring replication, larger sample sizes, and expanded research on chronic pain.

The insights from the studies included in this series converge on a central theme: the necessity of individualized, comprehensive, and integrative approaches to SCD pain management. A holistic framework that incorporates pharmacogenomics and TCM diagnostics to address the molecular drivers of bone pain and hemolysis is essential. Moreover, systemic barriers such as resource constraints and inequities in healthcare access must be addressed to ensure that these advancements benefit every patient. An improvement in patient outcomes and quality of life can be achieved through the integration of diverse therapeutic strategies and a comprehensive, patient-centered approach.

